# *Drosophila melanogaster* as an Animal Model for the Study of *Pseudomonas aeruginosa* Biofilm Infections *In Vivo*

**DOI:** 10.1371/journal.ppat.1002299

**Published:** 2011-10-06

**Authors:** Heidi Mulcahy, Christopher D. Sibley, Michael G. Surette, Shawn Lewenza

**Affiliations:** Department of Microbiology, Immunology and Infectious Diseases, University of Calgary, Calgary, Alberta, Canada; Stanford University, United States of America

## Abstract

*Pseudomonas aeruginosa* is an opportunistic pathogen capable of causing both acute and chronic infections in susceptible hosts. Chronic *P. aeruginosa* infections are thought to be caused by bacterial biofilms. Biofilms are highly structured, multicellular, microbial communities encased in an extracellular matrix that enable long-term survival in the host. The aim of this research was to develop an animal model that would allow an *in vivo* study of *P. aeruginosa* biofilm infections in a *Drosophila melanogaster* host. At 24 h post oral infection of *Drosophila, P. aeruginosa* biofilms localized to and were visualized in dissected *Drosophila* crops. These biofilms had a characteristic aggregate structure and an extracellular matrix composed of DNA and exopolysaccharide. *P. aeruginosa* cells recovered from *in vivo* grown biofilms had increased antibiotic resistance relative to planktonically grown cells. *In vivo*, biofilm formation was dependent on expression of the *pel* exopolysaccharide genes, as a *pelB::lux* mutant failed to form biofilms. The *pelB::lux* mutant was significantly more virulent than PAO1, while a hyperbiofilm strain (PAZHI3) demonstrated significantly less virulence than PAO1, as indicated by survival of infected flies at day 14 postinfection. Biofilm formation, by strains PAO1 and PAZHI3, in the crop was associated with induction of diptericin, cecropin A1 and drosomycin antimicrobial peptide gene expression 24 h postinfection. In contrast, infection with the non-biofilm forming strain *pelB::lux* resulted in decreased AMP gene expression in the fly. In summary, these results provide novel insights into host-pathogen interactions during *P. aeruginosa* oral infection of *Drosophila* and highlight the use of *Drosophila* as an infection model that permits the study of *P. aeruginosa* biofilms *in vivo*.

## Author Summary

*Pseudomonas aeruginosa* causes serious infections in people with compromised immune systems. Individuals with Cystic Fibrosis and hospital patients are particularly vulnerable to *P. aeruginosa* infections. This bacterium does not respond to many antibiotics, making these infections difficult to treat. *P. aeruginosa* can grow as free-floating planktonic cells or as microcolonies known as biofilms. The ability of *P. aeruginosa* to form biofilms is thought to contribute to their ability to cause chronic infections. The aim of this research was to develop a simple biofilm model of infection using the fruit fly (*Drosophila melanogaster*). The immune system of the fruit fly has similarities with the vertebrate innate immune system. Understanding how *P. aeruginosa* causes infections in *Drosophila* will aid in understanding virulence mechanisms in mammals. In this study we show that feeding *P. aeruginosa* to *Drosophila* results in a biofilm infection and biofilm infections induced expression of antimicrobial peptide immune response genes in the fly. Using fly survival as a measure of virulence we showed that biofilm infections were less virulent than non-biofilm infections. These results provide novel insight into host-pathogens interactions during *P. aeruginosa* infection.

## Introduction

*Pseudomonas aeruginosa* is an opportunistic pathogen capable of causing both acute and chronic infections in multiple hosts. The characteristics of acute and chronic infections caused by *P. aeruginosa* are quite distinct and are thought to be associated with co-ordinated expression of a select subset of virulence factors [1]. *P. aeruginosa* employs a number of strategies that promote chronic infection, including the ability to form microbial communities called biofilms [2]–[4]. Although biofilms have been extensively studied *in vitro*, the role of *P. aeruginosa* biofilms *in vivo* and how the host responds to biofilm infections, has been hindered by the lack of an appropriate model system. We sought to develop a simple biofilm model of infection that would allow the investigation of both the bacterial and host response during biofilm infections.

Biofilms are multicellular microbial communities encased in an extracellular matrix composed of extracellular DNA, multiple exopolysaccharides (EPS), proteins and lipids [5]–[7]. Typically, they display a complex three-dimensional structure and demonstrate increased resistance to antimicrobial compounds, environmental stresses and the host immune response [8], [9]. Exopolysaccharides have been shown to play a structural role in the biofilm as well as being involved in limiting antibiotic diffusion and protecting cells from antibody-mediated killing and phagocytosis by the host immune system [8], [10].

*P. aeruginosa* infects a wide variety of plants, insects and animals and there are a number of both plant and animal models used to examine bacterial pathogenesis [11]–[19]. *Drosophila melanogaster* (fruit fly) has gained popularity as a model organism for studying *P. aeruginosa* infections [20]–[27]. The reasons for this are as follows: (i) *D. melanogaster* displays evolutionary conservation of innate immune responses and NF-κB signaling cascades [28]; (ii) multiple genetic and molecular tools are available; (iii) the immune response can be measured in multiple ways e.g. clotting, phagocytosis, melanization and antimicrobial peptide (AMP) gene expression [28] and (iv) amenability to high throughput screening, relatively low cost and to date, no requirements for ethical approval.

One of the appealing features of *Drosophila* immunity is that many of its innate immune defenses display significant functional similarities with the vertebrate immune system (for review see [28]). These immune responses include the use of physical barriers, together with local and systemic responses. The fruit fly epithelia provide the first physical barrier to infection as well as a localized defense by production of AMP and reactive oxygen species (ROS.) Specialized hemocytes acts as phagocytes to engulf invading bacteria. Systemic production of AMPs occurs in the fat body, an organ metabolically similar to the human liver [28].

The *Drosophila melanogaster* genome encodes seven distinct classes of AMPs: the cecropins [29], diptericin [30], drosocin [31], defensin [32], drosomycin [33], metchnikowin [34] and attacin [35]. The fruit fly can discriminate between various classes of microorganisms [36], resulting in transcriptional activation of AMP genes. Depending on the pathogen associated molecular pattern (PAMP) of the infecting organism, distinct AMPs are induced upon infection by either the Toll or Imd pathway [28]. Although the induction of AMPs was initially thought to be specific to either the Toll or Imd pathway, there is ample evidence that the two pathways overlap [37]–[39].

Two *Drosophila* infection models have been described; the fly nicking or pricking [20] and fly feeding models [21], which are considered to resemble an acute or chronic infection, respectively. The nicking model consists of pricking flies with a needle dipped into bacterial culture and monitoring rapid fly killing within 1–3 days. In contrast, the fly feeding model results in a longer infection process with survival monitored up to 2 weeks postinfection. In this oral infection model, the main site of *P. aeruginosa* accumulation at 24h is the food storage organ, known as the crop [25]. In this study we hypothesized that the localized infection and slower killing kinetics observed following oral infection of *Drosophila* by *P. aeruginosa*, relative to the kinetics of nicking infection, was a result of the ability of *P. aeruginosa* to form microcolonies or biofilms in the *Drosophila* crop.

The aim of this study was to establish a relevant model for the study of *P. aeruginosa* biofilm infections *in vivo*. Additionally, we sought to investigate what bacterial genes were important in allowing *P. aeruginosa* to develop biofilm infections in this model and to identify the host AMP response to both biofilm and non-biofilm infections. The development of a biofilm infection model for studying both the bacterial and host response during infection has the potential to significantly increase our understanding of the relationship between biofilms and the host during infection.

## Results/Discussion

### *P. aeruginosa* forms biofilm in the *Drosophila* crop

Using the fly oral model of infection, 1–3 day old male flies were infected with *P. aeruginosa*. As we have been previously published, the predominant site of *P. aeruginosa* accumulation at 24 h was the food storage organ known as the crop [25] with bacteria moving from the crop into other areas of the gut over time (Figure S1). The presence of bacterial cells in areas of the gut outside of the crop is consistent with what has been shown by other groups [40], [41].

We used *P. aeruginosa* PAO1pCHAP6656 to visualize *P. aeruginosa* colonization *in vivo* as these cells produced mCherry as an outer membrane-anchored lipoprotein. PAO1pCHAP6656 was used as it has an easily identifiable membrane-staining pattern [4], [42] and can be used to differentiate true bacterial cells from red autofluorescence in the tissue of the *Drosophila* crop [25]. The pCHAP6656 plasmid [42] encodes for gentamicin resistance.

**Figure 1:**
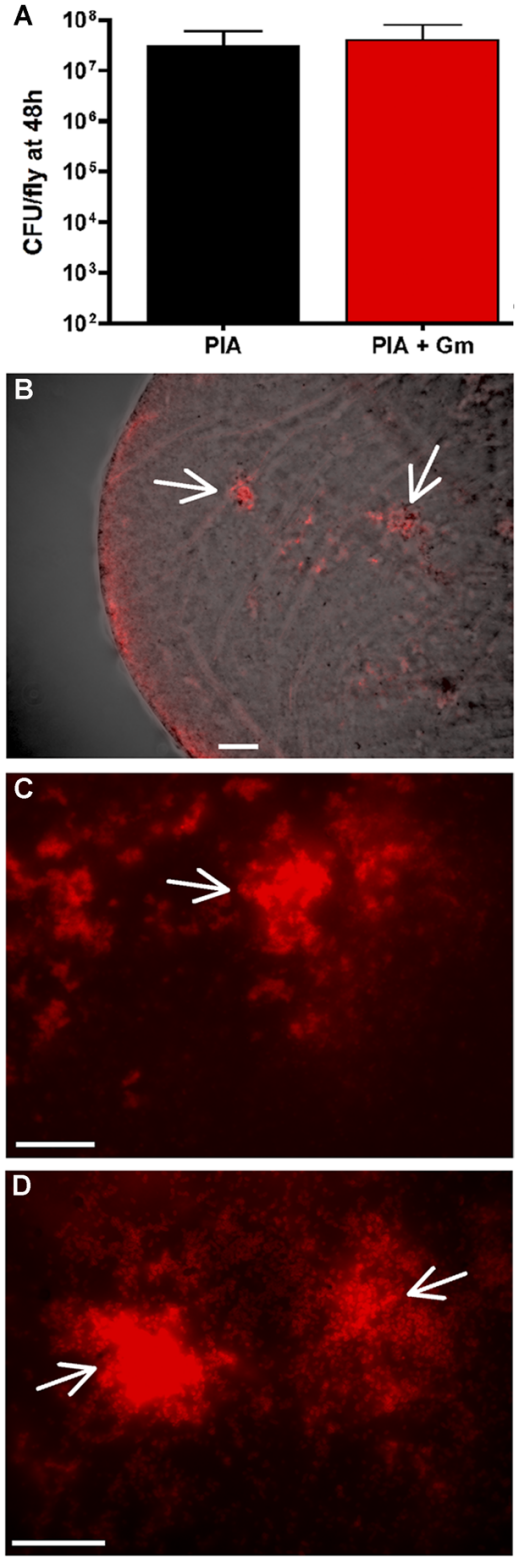
PAO1 (pCHAP6656) infection of the *Drosophila* crop. (A) Plating of bacteria recovered from infected flies on *Pseudomonas* isolation agar (PIA) +/− Gm 30 (µg/ml) indicated that pCHAP6656 was not lost up to 48 h postinfection. (B) Merged image of brightfield and red fluorescence images from PAO1-infected crops (40x). Red fluorescence images of infected crops at (C) 63x and (D) 100x objectives. White arrows indicate the presence of large bacterial aggregates. Scale bars indicate 200 µM. At least three infected crops were examined from three separate infections and representative images are shown.

To ensure that this was a suitable system for use *in vivo*, in the absence of antibiotic selection, we monitored plasmid maintenance up to 48 h postinfection. Plating of bacteria recovered from infected flies indicated that pCHAP6656 was maintained up to 48 h postinfection (Figure 1A). At 24 and 48 hours postinfection, flies were sacrificed and crops were surgically removed for microscopic analysis. Imaging of dissected crops indicated that bacteria localized to the periphery of the crop (Figure 1B) and that large aggregates (50–250 µM) or microcolonies were visible at 24 h (Figure 1B–D). These microscopic observations showed *P. aeruginosa* was present in the *Drosophila* crop, at high cell densities (∼3×10^7^ CFU/crop, Figure 1A) and organized in microcolonies, indicating the presence of biofilms as early as 24 h postinfection of *Drosophila*.

### *In vivo* biofilms are composed of *P. aeruginosa* microcolonies, EPS and DNA, that form a characteristic honeycomb-like shape

**Figure 2:**
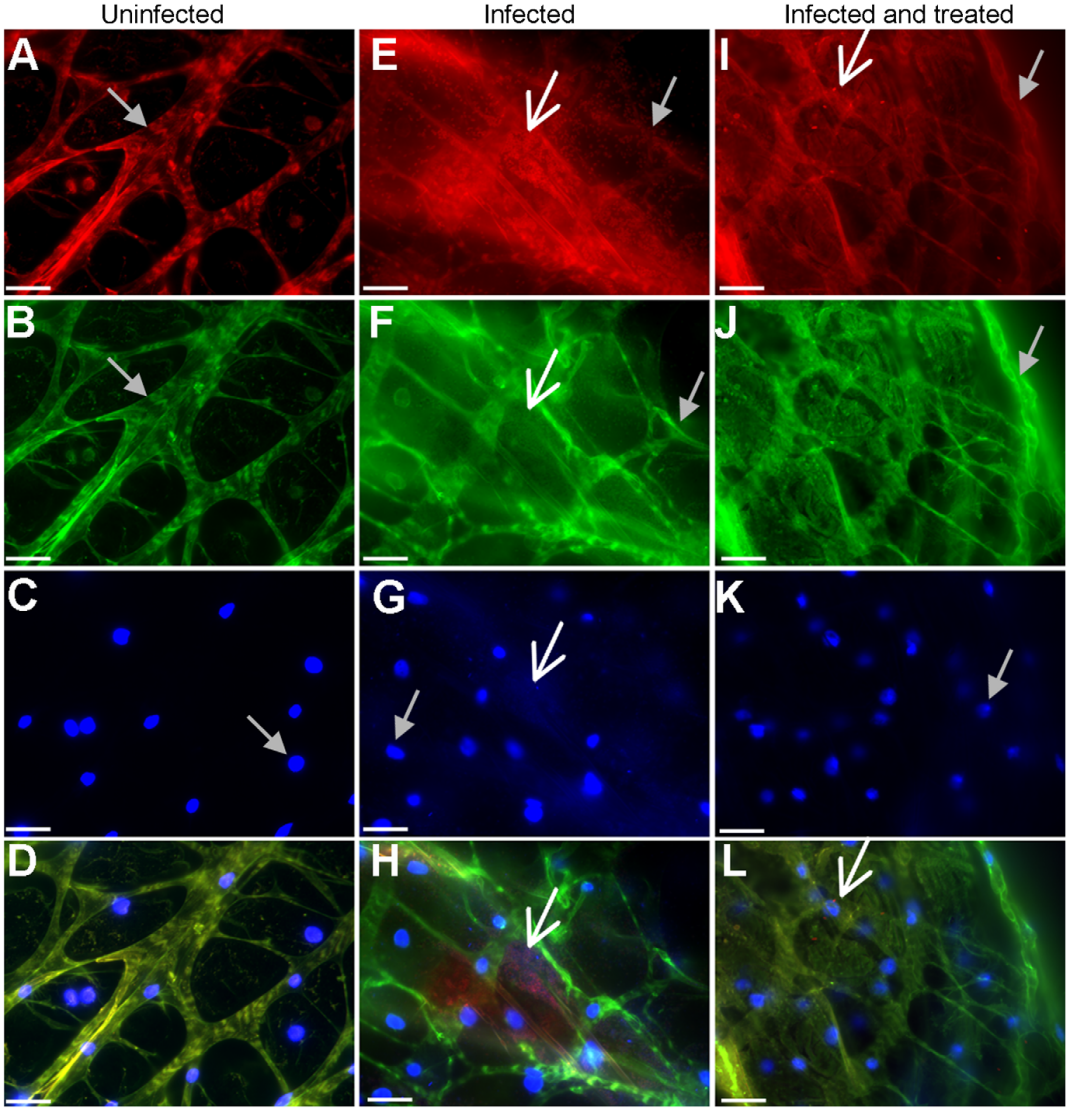
*In vivo P. aeruginosa* biofilms stain positively for EPS and DNA. (A) Red autofluorescence, (B) green autofluorescence, (C) DAPI-stained nuclei (all indicated by grey arrow) and (D) merge image of uninfected *Drosophila* crop. (E) Aggregative red fluorescent PAO1pCHAP6656 (white arrow) along with red autofluorescence (grey arrow), (F) green fluorescent EPS (white arrow) staining and autofluorescence (grey arrow), (G) DAPI staining of bacteria (white arrow) and *Drosophila* nuclei (grey arrow) and (H) merge image of PAO1-infected crops. DNAse and cellulase treatment of *P. aeruginosa*-infected crops. (I) Non-aggregative red fluorescent PAO1pCHAP6656 (white arrow) along with red autofluorescence (grey arrow), (J) autofluorescence (grey arrow) and lack of EPS staining with FITC-labeled HHA lectin. (K) DAPI staining of *Drosophila* nuclei (grey arrow) and absence of bacterial DNA staining and (L) merge image of DNAse and cellulase treated PAO1 in infected crops (white arrow). Scale bars in indicate 100 µM. At least three infected crops were examined from two separate infections and representative images are shown.

*In vitro* grown biofilms are characterized by an extracellular biofilm matrix composed of DNA and EPS [5]–[7]. To determine if the *P. aeruginosa* microcolonies observed in *Drosophila* displayed similar characteristics to those of *in vitro* biofilms, crops were stained for EPS and DNA. We exploited the red and green autofluorescence of the crop (Figure 2A–B), while simultaneously visualizing red fluorescent PAO1pCHAP6656 (Figure 2E), green fluorescent EPS (Figure 2F) and blue fluorescent DNA (Figure 2G). The gross morphology of uninfected crops was compared to PAO1pCHAP6656-infected crops. Uninfected crops had clearly defined muscular fibers and cellular structures (Figure 2A–D). PAO1pCHAP6656-infected crops (Figure 2E–H) demonstrated a loss in the musculature, a blurring of the fibers and an overall lack of the organized structure visible in uninfected crops (Figure 2A–D). FITC conjugated Hippeastrum hybrid Lectin (HHA) [43] (green fluorescence) was used to label exopolysaccharide present in the microcolonies (Figure 2F). DAPI (blue fluorescence) was used to visualize DNA, which is present in the nuclei of *Drosophila* epithelial cells lining the crop (Figure 2C), in bacterial cells (Figure 2G) and as extracellular DNA surrounding bacterial cells and in the biofilm matrix (Figure 2G). PAO1pCHAP6656 (red) was visible as aggregates in the crop (Figure 2E). An overlap between bacteria (red), EPS (green) and DAPI (blue) was visible (Figure 2H) suggesting that bacterial aggregates stain positively for EPS and DNA. Red and green autofluorescence of the crop itself was also observable (Figure 2A–D). DAPI was used as the stain of choice for DNA. Use of other green or red DNA stains such as TOTO-1 or Sytox red, resulted in very high background due to autofluorescence in the crop. These data indicated that at 24 h postinfection, *P. aeruginosa* biofilms can be visualized in dissected crops of infected flies and these biofilms stain positively for DNA and EPS, two major characteristics of an *in vitro* biofilm.

To show that DNA and EPS were important biofilm components *in vivo*, infected crops were DNAse- and cellulase-treated prior to EPS and DNA staining (Figure 2I–L) and compared to uninfected (Figure 2A–D) and PAO1pCHAP6656-infected crops without DNAse and cellulase treatment (Figure 2E–H). Aggregates of bacteria, which stained positively for DNA and EPS were visible in infected crops (Figure 2E–H). No aggregates were detected in PAO1pCHAP6656-infected crops treated with DNAse and cellulase, indicating that bacterial biofilms were dissolved following enzymatic treatment (Figure 2I–L). DNAse and cellulase treatment of uninfected crops had no effect on crop structure (data not shown).

**Figure 3:**
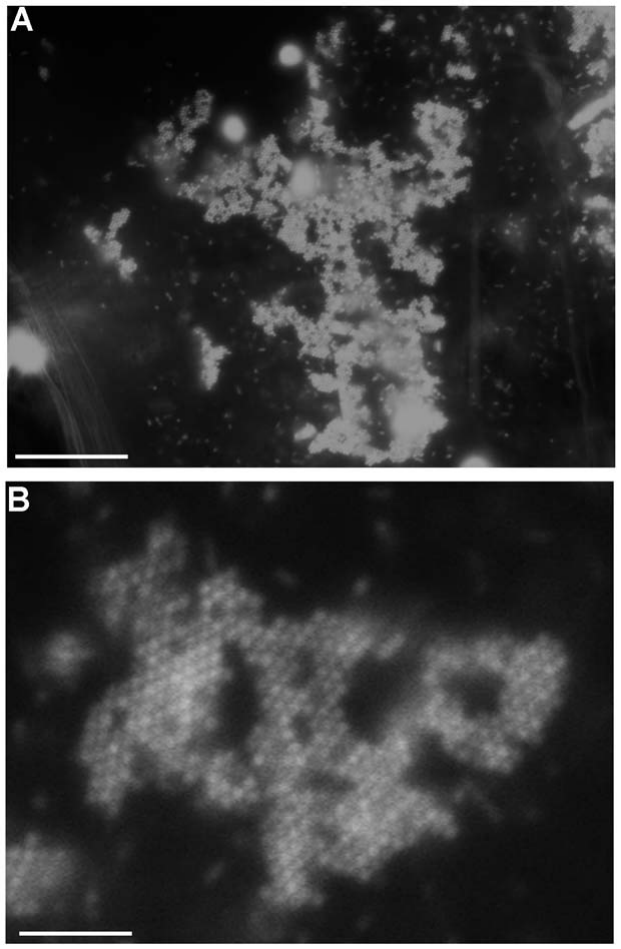
Visualization and staining of *in vivo* microcolonies in the *Drosophila* crop. (A) DAPI-stained bacterial cells (100X) and (B) digitally zoomed images (4.4X) of DAPI stained microcolonies in the crop, demonstrating a honeycomb-like structure (white arrow). Scale bars in A indicate 200 µM. Scale bars in B indicate 45.4 µM. At least three infected crops were examined from three separate infections and representative images are shown. Honeycomb-like structures were visualized in 2 out of every three PAO1-infected crops examined.

On closer inspection of DAPI-stained bacteria in the crop (digital zoom of 4.4X), we observed that the bacteria in the microcolonies were organized into characteristic patterns or clusters (Figure 3). Cells that we previously observed to be positive for DNA and EPS staining (Figure 2A–D) were organized into a cluster of hexagonal bacterial colonies. These structures were found in two orientations, with bacteria lined up side-to-side and stacked one on top of the other or with the pole to pole length of the bacterial cells visible, and each cell attached side-to-side (Figure 3). Because of the size of each of the hexagons, approximately 6–8 µM, we predict that each hexagon is made up of approximately 7 to 15 bacterial cells. This is consistent with what has been observed for mature honeycomb structures produced by *S. epidermidis* [44]. These honeycomb-like shaped microcolonies were not firmly attached to the epithelial cell surface but were floating inside the enclosed fly crop (Video S1).

Microbial species, including *Sinorhizobium meliloti*, *Rhizobium leguminosarum* [45], [46], *Staphlococcus epidermidis and P. aeruginosa* [44] were previously shown to form complex biofilm structures, organised in honeycomb- and veil-like patterns. Honeycomb structures are one of the most densely packed structures found in nature and similar to what is observed with bee honeycombs [47], it is predicted that these structures enable close packing together of cells with the least amount of matrix components, including energy-expensive EPS. To our knowledge, bacterial microcolonies that resemble honeycomb structures have not previously been visualized *in vivo* in an animal model and highlight the use of *Drosophila* as an infection model amenable to microscopic analysis of infected tissues.

### *P. aeruginosa* biofilm infection results in loss of integrity of the fly crop structure

**Figure 4:**
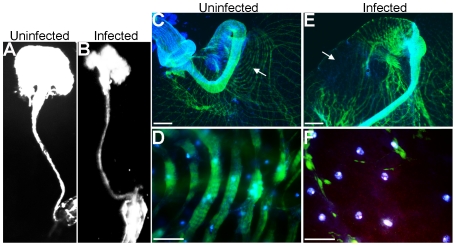
Crop integrity in response to *P. aeruginosa* infection. (A) The macroscopic structure of (A), uninfected and (B), PAO1pCHAP6656-infected *Drosophila* crops (Olympus OV100 intravital observation system). Merged fluorescent image of phallodin 488-stained actin (green) and DAPI-stained nuclei (blue) in uninfected crops using (C) 10x and (D) 63x objectives. PAO1pCHAP6656-infected crops (red) at (E) low and high (F) magnification. Scale bars in C and E indicate 400 µM; scale bars in D and E indicate 100 µM. White arrows in C and E indicate the area of the crop where higher magnification images were taken. At least five infected crops were examined from two separate infections and representative images are shown.

To investigate the potential for detrimental consequences of *P. aeruginosa* biofilm infections on host tissue we examined the architecture of infected fly crops during a biofilm infection. Excised crops were visualized macroscopically for gross changes in shape and size at 10X magnification (Figure 4A and B). Brightfield imaging of uninfected (Figure 4A) and PAO1-infected (Figure 4B) crops indicated significant changes in the size and gross morphology of the crop in response to infection. Infected crops were smaller in size, softer in texture and were more sensitive to breaking apart upon handling (data not shown). This observation is consistent with tissue damage however it is also possible that a smaller softer crop may be as a result of an empty crop as *P. aeruginosa* infection may interfere with the ability of *Drosophila* to feed and drink.

To investigate the morphological structure of the musculature of the crop in greater detail in the presence and absence of biofilm infection, F-actin staining using Phallodin 488 was performed. *Drosophila* nuclei were counterstained with DAPI and crops examined by fluorescence microscopy. The musculature of uninfected crops consisted of wide ribbons of circular muscles covering the crop wall with a intricate network of branched and interconnecting fibers (Figure 4CD) This architecture was severely compromised or absent in infected crops (Figure 4EF). *P. aeruginosa* (red) localized predominantly to the crop edge, which was where the most disorganised actin staining was detected, including depolymerisation and degradation of actin filaments (Figure 4F).

### Expression of EPS is essential for *in vivo* biofilm formation

**Figure 5:**
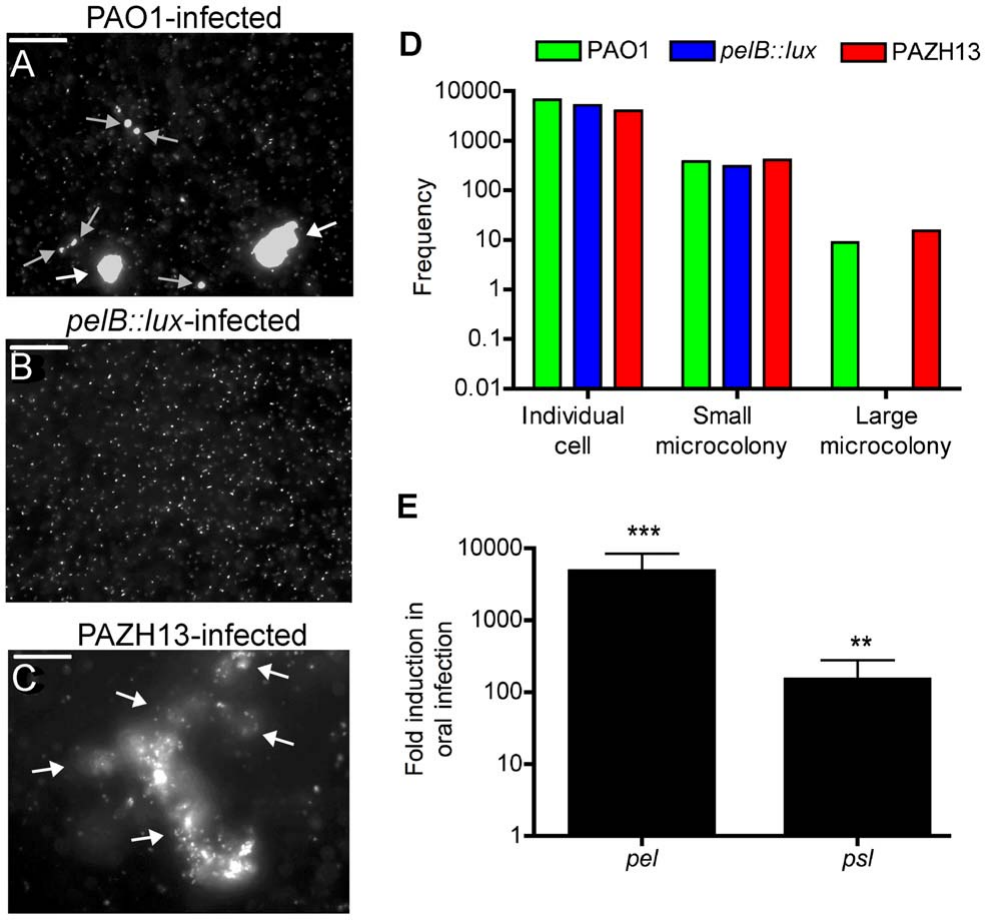
The role of Pel EPS during *in vivo* biofilm formation in *Drosophila.* Representative images of *P. aeruginosa* pCHAP6656-infected crops. (A) PAO1pCHAP6656-infected crops contain individual bacterial cells, a number of small microcolonies (grey arrows) and two large microcolonies (white arrows). (B) *PelB::lux*pCHAP6656-infected crops contain individual bacterial cells and no small or large microcolonies. (C) PAZH13pCHAP6656-infected crops contain some individual bacterial cells, and five large microcolonies (white arrows). At least 3 infected crops were examined for each strain. Scale bar equals 100 µM. (D) Quantitative analysis of microcolony formation in response to infection with PAO1 and relevant mutant strains. At least 3 infected crops were examined for each strain. Data presented is the frequency of individual bacterial cells, small or large bacterial microcolonies in a total of 12 fields of view. (E) Expression of *pel* and *psl* EPS genes during oral infection relative to acute infection. Values are mean +/− SEM from triplicate qRT-PCR experiments on RNA isolated from two independent *Drosophila* infections.

We examined *in vivo* biofilm phenotypes of *P. aeruginosa* mutants known to exhibit altered EPS production and biofilm formation phenotypes *in vitro.* The *pelB::lux* mutant [48] is defective for biofilm formation *in vitro*, as mutants in the *pel* operon are known to have decreased EPS production and biofilm formation [49], while strain PAZHI3 (a mutant in the posttranscriptional regulatory protein RsmA) displayed increased production of both *pel* and *psl* EPS (Figure S2) and is a hyperbiofilm former [50]. Flies were infected with PAO1, *pelB::lux* or PAZHI3, all carrying pCHAP6656, and 24 h postinfection crops were excised and examined for the presence of microcolonies. Microscopy was performed on PAO1pCHAP6656, *pelB::lux*pCHAP6656, and PAZH13pCHAP6656-infected crops. Twelve fields of view were captured, from a minimum of 3 crops infected with each strain (Figure 5A–C), for quantification of microcolony formation (Figure 5D). Image analysis (ImageJ) was performed to differentiate and count the frequency of individual cells, as well as small and large microcolonies (Figure 5A) (See materials and methods for additional information). Large microcolonies (>20 cells, Figure 5A,C) were present only in PAO1- or PAZHI3-infected crops and were absent from *pelB::lux*-infected crops (Figure 5B). PAZHI3-infected crops had more microcolonies (n = 15) present than those seen in PAO1-infected crops (n = 9) (Figure 4D). Furthermore, the large microcolonies (categorized as those microcolonies consisting of >20 cells) observed in PAZH13-infected crops were significantly larger (p<0.001) in size (approx 17-fold) that those microcolonies observed in PAO1-infected crops indicating the hyper-biofilm features of PAZHI3 detectable *in vitro* were also observed *in vivo*. No large microcolonies were detected in *pelB::lux*-infected flies (Figure 4B).

To further confirm the importance of EPS during oral *Drosophila* infection, qRT-PCR was used to measure expression of *pel* and *psl* during infection. *Psl* expression was significantly induced, approximately 150 fold (p<0.01). *Pel* expression was highest during oral infection of flies, induced approximately 2200 fold (p<0.001), relative to acute infection of flies (Figure 5E). These data indicate that Pel may play a more important role during oral infection of *Drosophila*, since it is more highly expressed. These data highlight the importance of Pel EPS as a biofilm matrix component for the establishment and/or maintenance of biofilms *in vivo*, in addition to its well-characterized importance for attachment and maturation, during the early and later stages of biofilm formation *in vitro* [51]–[53].

### Non-biofilm forming strains disseminate at a faster rate than biofilm forming strains following oral infection

**Figure 6:**
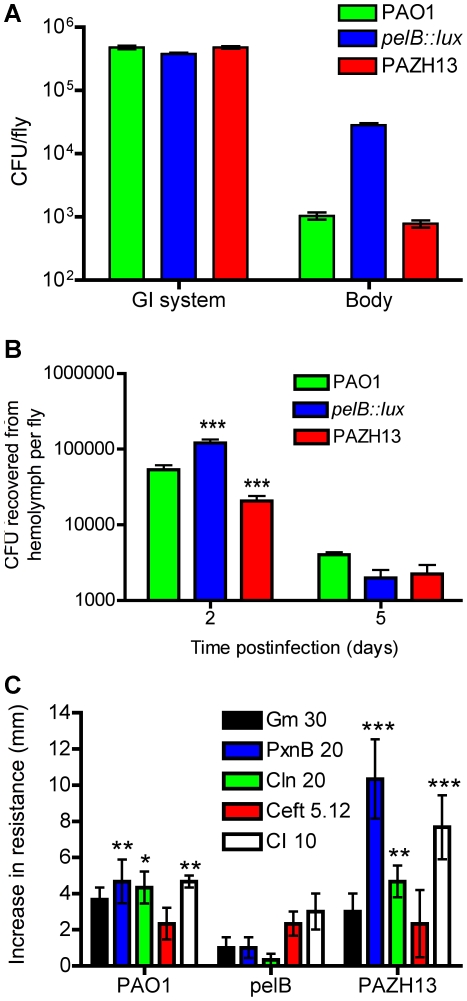
*In vivo* localization and antibiotic resistance profiling of biofilm and non-biofilm infections. (A) Localization of bacteria in the fly 5 days postinfection. The GI tract including the crop, was dissected out, crushed and plated on PIA agar to determine CFU per GI tract/fly. The remainder of the fly body, including the head, was crushed separately and plated on PIA to determine CFU/rest of body per fly. (B) The number of CFU recovered from *Drosophila* hemolymph 2- and 5-days postinfection with PAO1, PAZH13 or *pelB::lux*. Two biological replicate experiments were performed, each containing 20 *Drosophila*, and values represented are mean +/−SEM. (C) Antibiotic resistance profiling of biofilm and non-biofilm infections. Increase in antibiotic resistance, as measured by zone of inhibition in disk diffusion assay, in *P. aeruginosa* strains recovered for *Drosophila* after oral infection relative to planktonic cultures. Antibiotic concentration indicated in µg/ml. Two biological replicate experiments were performed in triplicate and mean +/− SEM is shown. * p<0.05, ** p<0.01, ***p<0.001.

We hypothesized that biofilm forming and non-biofilm forming strains would differ in their ability and/or timing to disseminate and that ultimately the kinetics of bacterial dissemination may play a role in fly survival. Initial experiments were performed to compare *in vivo* localization of PAO1, *pelB::lux* and PAZHI3 strains in infected flies. Results of viable plate counts indicated a slightly lower bacterial load was recovered from the GI tract of *pelB::lux*-infected flies (3.8×10^5^±1.82×10^4^ CFU/fly; mean ± SEM), compared to that of PAO1-infected flies (4.8×10^5^±2.95×10^4^ CFU/fly). There was a corresponding increase in the number of viable *pelB::lux* bacteria (2.8×10^4^±1.83×10^3^ CFU/fly), recovered from the fly body, excluding the GI system, compared to that of PAO1-infected flies (1.03×10^3^±1.38×10^2^ CFU/fly) 5 days postinfection (Figure 6A). Similar numbers of bacteria were isolated from the GI system or fly body of PAZH13-infected flies compared to PAO1-infected flies. To provide evidence of altered dissemination between biofilm and non-biofilm forming strains, hemolymph was recovered from infected flies at day 2 and day 5 postinfection. The *pelB::lux* mutant was present in the hemolymph at significantly higher numbers than PAO1 or PAZH13 at two days postinfection, while no significant difference in dissemination was observed five days postinfection (Figure 6B). Previous studies have shown that *pelA* mutants demonstrated increased rates of swarming motility [49], which in combination with reduced biofilm formation and may contribute to the increased rate of dissemination observed during infection of *Drosophila* 2 days postinfection. The fact that significantly increased numbers of *pelB::lux* are observed in the hemolymph 2 days postinfection relative to PAO1, while no significant difference is observed 5 days postinfection, suggests that upon detection by the host immune system in the hemolymph, *pelB::lux* bacteria are unable to persist or are cleared by the immune system.

### *P. aeruginosa* recovered from biofilm infections *in vivo* have increased resistance to antimicrobials

Resistance to antimicrobials is a general feature of all biofilms. We hypothesized that PAO1 recovered from a biofilm infection of *Drosophila* would display increased resistance to antimicrobials. Antimicrobial sensitivities were compared in PAO1 directly recovered from flies relative to PAO1 planktonic cultures or PAO1 planktonic cultures exposed to pulverized fly tissues, termed “mock-infected” PAO1. Identical bacterial inocula from these three conditions were swabbed onto *Pseudomonas* Isolation Agar (PIA) and antimicrobial sensitivity was measured by disk diffusion. Antimicrobial resistance of PAO1 recovered from mock-infected cultures was not significantly different relative to the resistance profiles of PAO1 recovered from planktonic cultures (data not shown). PAO1 directly recovered from infected flies had significantly increased resistance to polymyxin B, colistin, and ciprofloxacin, but not to gentamicin or ceftazidime relative to planktonic PAO1 cultures (Figure 6C). PAZHI3 recovered from infected flies was also significantly more resistant to polymyxin B, colistin, and ciprofloxacin than planktonic PAZHI3 (Figure 6C). In contrast, antimicrobial resistance profiles of the *pelB::lux* mutant (which failed to form biofilms *in vivo*) were not significantly different in cells directly recovered from infected flies relative to planktonic or mock-infected cells (Figure 6C).

Polymyxin B and colistin are cationic AMPs: short, amphipathic peptides that bind to and disrupt both the outer and cytoplasmic membranes resulting in bacterial cell death [54]. Ciprofloxacin is a member of the fluoroquinolone drug class which inhibits DNA gyrase and hence DNA replication. The increased antibiotic resistance phenotype of *P. aeruginosa* recovered from the flies compared to planktonic cultures, is analogous to the increased resistance observed in *in vitro* biofilm populations compared to planktonic cultures [4], [55].

### *Drosophila* infected with biofilm and non-biofilm forming *P. aeruginosa* have altered survival kinetics

**Figure 7 ppat-1002299-g007:**
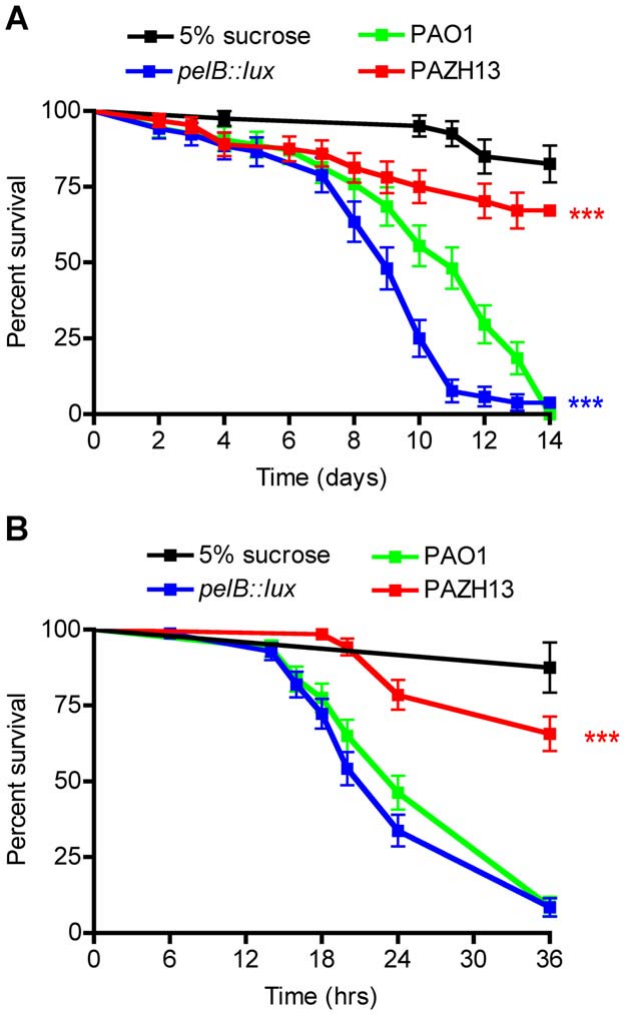
Kaplan-Meier survival curves post *P. aeruginosa* infection. Survival curves of (A) oral and (B) acute infection with PAO1, *pelB::lux*, PAZH13 or 5% sucrose control. Experiments were performed at least 3 times each with a minimum of 80 flies and representative curves (mean +/− standard deviation) are shown. *** p<0.001.

To assess the comparative abilities of biofilm and non-biofilm forming *P. aeruginosa* strains for their ability to cause disease in *Drosophila*, we monitored fly survival over 14 days in response to oral infection. The non-biofilm forming *pelB::lux* mutant was significantly more virulent compared to PAO1, having a significantly increased rate of *Drosophila* killing (Figure 7A). In contrast, hyperbiofilm-forming PAZHI3 demonstrated significantly reduced virulence compared to PAO1, as indicated by a greater survival of infected flies up to 14 days postinfection (Figure 7A). There was no difference in the bacterial load (CFU) in biofilm and non-biofilm infected flies (data not shown). PAO1 mutants in *psl* showed similar killing kinetics to PAO1-infected flies (Figure S3) indicating that Pel EPS contributes to pathogenesis during infection of *Drosophila* while Psl EPS does not. Previous *in vitro* studies have indicated that both Pel and Psl are important in *P. aeruginosa* biofilm formation [6], [52] and that Pel EPS also contributes to antibiotic resistance [56]. Our data highlight a unique role for Pel EPS in *P. aeruginosa* biofilm formation *in vivo*, as well as a role in dissemination and virulence.

The production of Pel EPS and biofilm formation inversely correlated with virulence and the ability of *P. aeruginosa* to cause death in *Drosophila* after oral infection (Figure 7A). To determine if EPS production also affected the outcome of acute *P. aeruginosa* infections, *Drosophila* killing kinetics were compared in male flies nicked in the thoracic region, with the relevant *P. aeruginosa* strains, up to 36 h. Pel production was not found to be important factor during acute infection as PAO1 and *pelB::lux* infections resulted in similar killing kinetics in acutely-infected *Drosophila* up to 36 h postinfection (Figure 7B). PAZHI3 was attenuated for virulence during acute infection, similar to what was seen for oral infection (Figure 7). Reduced killing of *Drosophila* by PAZHI3 is similar to reduced virulence previously observed for PAZHI3 in a mouse model of acute infection [50].

### Biofilm infections induce AMP gene expression

**Figure 8:**
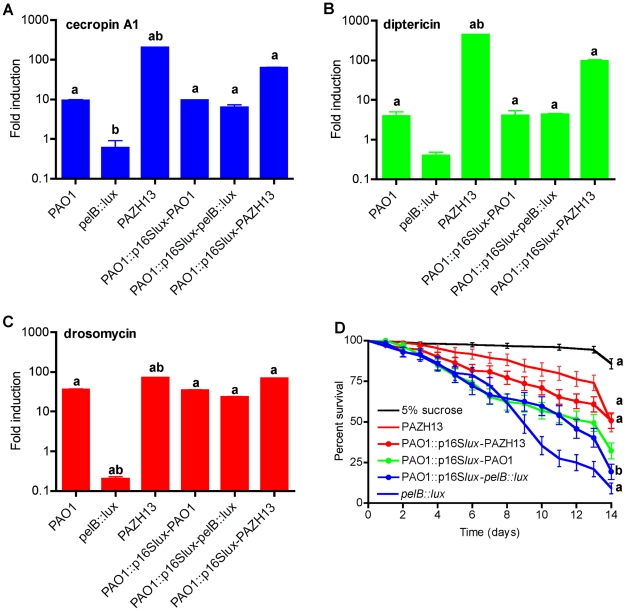
Biofilm infections induce antimicrobial peptide gene expression in *Drosophila*. Real time RT-PCR analysis of (A) cecropin A1 (B) diptericin and (C) drosomycin following oral infection with PAO1, *pelB::lux*, PAZH13 or following oral co-infection with a 1∶1 ratio of PAO1-PAO1p16S*lux*, PAO1p16S*lux*-*pelB::lux* or PAO1p16S*lux*-PAZH13. For co-infection experiments (last 3 bars) the strains used for each infection are listed, separated by a hyphen. The levels of AMP gene expression was represented as fold change relative to uninfected flies. Values are mean +/− SEM from triplicate qPCR experiments on RNA isolated from two independent *Drosophila* infections. a, significant fold change (p<0.05, ANOVA) relative to uninfected flies; b, significant fold change (p<0.05, ANOVA) relative to PAO1-infected flies. (D) Kaplan-Meier survival curves of *Drosophila* following oral co-infection with a 1∶1 ratio of PAO1-PAO1p16S*lux*, PAO1p16S*lux*-*pelB::lux*, PAO1p16S*lux*-PAZH13 and relevant controls. Experiments were performed at least 3 times each with a minimum of 80 flies and representative curves (mean +/− standard deviated) are shown. a, significant difference (p<0.05, ANOVA) relative to PAO1-PAO1p16S*lux*-infected flies (green); b, significant difference (p<0.05, ANOVA) relative to *pelB::lux*-infected flies.

To monitor the AMP response to biofilm and non-biofilm infections in *Drosophila,* we assessed the expression of the AMP genes cecropin A1, diptericin and drosomycin using qRT-PCR during oral infection with PAO1, *pelB::lux* and PAZHI3 (Figure 8A–C). As no difference in killing kinetics were observed between flies acutely infected with biofilm forming PAO1 and non-biofilm forming *pelB::lux,* AMP gene expression was not monitored following acute infection.

PAO1 oral infection induced the expression of cecropin A1, diptericin and drosomycin between 4- and 36-fold relative to uninfected flies (Figure 8A–C). Increased gene expression was also detected in PAZHI3-infected flies at levels between 72- and 446-fold. While PAZH13 is hyperbiofilm former *in vitro* [50] and *in vivo* (Figure 5C), it is also a pleiotrophic mutant [57]–[61]. Thus, while there is a correlation between biofilm formation and increased AMP expression, we cannot rule out the possibility that the higher levels of AMP induction seen in response to PAZH13 infection may not be solely attributable to increased biofilm formation. In response to *pelB::lux* infection, we observed lower expression of all three AMP genes, between 1.6- and 5-fold, compared to uninfected flies. Suppression of AMP gene expression is thought to be one of the main mechanisms whereby commensal bacteria fail to elicit an immune response in the host [62]. However, virulent strains of *P. aeruginosa* have also been documented to suppress AMP gene expression and the *Drosophila* immune response during an acute infection [24]. In this study, decreased expression of AMP gene expression by the *pelB::lux* mutant (Figure 8) appears to be associated with increased fly death following oral infection as flies die at a significantly faster rate compared to those infected with the biofilm forming Pel positive strains PAO1 or PAZH13 (Figure 7A). While this study does not demonstrate active suppression, it is possible that increased fly mortality post oral infection resulted from decreased expression of AMP gene expression in the fly and/or a more rapid (within 2 days) dissemination of *pelB::lux* to the hemolymph, resulting in systemic infection and fly death. However in addition to difference in localization of *pelB::lux* (Figure 6A), it may also be that *pelB::lux* is more toxic, eliciting pathological changes in *Drosophila* resulting in more rapid death.

As EPS can be a cell-surface or secreted product, we hypothesized that co-infection of *Drosophila* with a 1-1 mixture of *P. aeruginosa* wildtype and *pelB::lux* would restore AMP gene expression and killing, similar to levels observed in orally PAO1-infected flies. In these experiments, PAO1::p16S*lux* [63] was used instead of PAO1 as the wildtype strain as bacterial load and AMP gene expression did not differ significantly in PAO1::p16S*lux*-infected flies compared to PAO1-infected flies (data not shown). Use of PAO1::p16S*lux* allowed us to differentiate between wildtype and mutant strains for quantitative bacteriology using erythromycin resistance in PAO1::p16S*lux* as the differentiating marker. Relative to uninfected flies, AMP gene expression was measured in flies co-infected with PAO1::p16S*lux* and PAO1, *pelB::lux* or PAZHI3. There was no significant difference in AMP gene expression following co-infection with PAO1::p16S*lux* and PAO1 (Figure 8A–C). Co-infection with PAO1::p16S*lux* and *pelB::lux* resulted in induction of 6.3-, 4.3-, and 23-fold for cecropin A1, diptericin and drosomycin, respectively (Figure 8A–C), induction levels similar to those observed for wildtype infections. For PAO1::p16S*lux* and PAZHI3 co-infected flies, AMP genes were induced at levels between 62 to 97 fold (Figure 8A–C). These data indicated that co-infection of *pelB::lux* and PAO1 restored AMP gene expression to levels similar to those observed in PAO1-infected flies. In all co-infection experiments, quantitative bacteriology was performed at T_0_, T_24_ and T_120_ (hours) to ensure that the bacterial load was at a ratio of approximately 1-1 at the initial stage of infection (T_0_), at the time of RNA extraction (T_24_), and at later time points during infection (T_120_) (Figure S4). No significant differences were observed in the growth of different bacterial strains in *Drosophila* following co-infection at any of the time points investigated, indicating that *pelB::lux* or PAZH13 mutants were not altered in their ability to compete with PAO1 for colonization during infection of *Drosophila*.

*Drosophila* survival was also monitored following co-infection experiments. Co-infection of flies with *pelB::lux* and PAO1::p16S*lux* resulted in significantly increased fly survival relative to *pelB::lux-*infected flies, increasing fly survival to levels similar to those seen during wildtype infection (PAO1 and PAO1::p16S*lux*) (Figure 8D). Co-infection of flies with PAZH13 and PAO1::p16S*lux* had no significant effect on fly survival with flies dying at similar rates regardless of whether they were infected with PAZH13 alone or co-infected with PAZH13 and PAO1::p16S*lux*. Single infection with PAO1::p16S*lux* or PAO1, or co-infection with PAO1 and PAO1::p16S*lux*, had similar killing kinetics (data not shown).

Prior to this study, it was not known if the fly immune system responded differently to biofilm and non-biofilm forming bacteria. Drosomycin expression is regulated through the Toll pathway [64]; Diptericin is regulated via the Imd pathway [65] and both pathways overlap to regulate cecropin A1 expression [66]. Our data indicates that both of the central immune pathways in *Drosophila* are activated in response to biofilms. In addition this data indicates that it is Pel positive biofilms, and possibly Pel EPS itself, that may act as a specific host immune signal inducing AMP gene expression in *Drosophila* as a *psl* mutant has no effect on *Drosophila* killing (Figure S3). Future work will focus on identifying the specific bacterial components involved in AMP gene expression and other host signalling pathways in response to Pel and Psl positive biofilms and non-biofilm *P. aeruginosa* infections.

In the *Drosophila* oral infection model, our data suggests that Pel positive biofilms induced AMP gene expression in the fly. Although biofilm infections induce AMP gene expression (Figure 8A–C), biofilm-forming bacteria isolated from fly crops postinfection are more resistant to the AMPs polymyxin B and colistin than those recovered from planktonic cultures (Figure 6). Bacterial Pel EPS may be a cue to the host to increase AMP gene expression thus serving to slow dissemination of the bacteria, and in this way slow systemic infection which would rapidly kill the host. On the other hand, EPS may also induce inflammation in the crop/GI system resulting in a localized damage to the host. Strains incapable of forming Pel positive biofilms *in vivo* resulted in a decreased AMP response but disseminated earlier, resulting in a systemic infection associated with faster host killing. These interpretations are supported by the *Drosophila* survival data obtained from co-infection experiments, where co-infection of flies with *pelB::lux* and PAO1 significantly increases *Drosophila* survival compared to infection with *pelB::lux* alone.

### Biofilm infections do not alter kinetics of subsequent acute infection but modify fly survival in response to subsequent oral challenge

**Figure 9:**
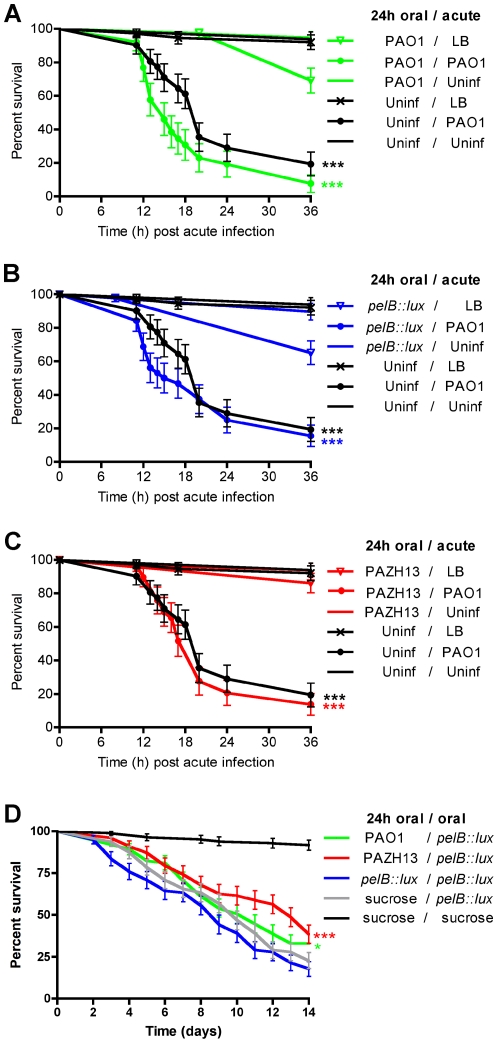
Kaplan-Meier survival curves of *Drosophila* orally infected (feeding) for 24h followed by subsequent acute (nicking) or secondary oral infection. Survival following oral infection with (A) PAO1, (B) *pelB::lux* or (C) PAZH13 and relevant controls followed by acute infection with PAO1 or relevant controls. (D) *Drosophila* survival following oral infection with PAO1, *pelB::lux*, PAZH13 or uninfected (sucrose control) followed by oral infection with *pelB::lux* or uninfected (sucrose control). Strain name preceding the forward slash "/" indicates the strain or uninfected sucrose control used for oral infection of *Drosophila*; the strain name following the forward slash "/" indicates the strain or nicked (LB) or uninfected sucrose controls used for subsequent acute or secondary oral infection of *Drosophila*. Experiments were performed at least twice, each with a minimum of 100 flies and representative curves (mean +/- standard deviated) are shown. * p<0.05, ***p<0.001.

It has previously been shown that *P. aeruginosa* eludes host defenses by suppressing AMP gene expression in a *Drosophila* model of acute infection [24]. This study also demonstrated that infection with a less virulent *P. aeruginosa* strain resulted in immune potentiation and protected flies from subsequent acute infection with a more virulent *P. aeruginosa* strain [24]. To determine if oral infection, biofilm formation and induction of AMPs in *Drosophila* could alter the kinetics of fly survival following subsequent acute infection, we performed the following experiment. Male flies were orally infected with PAO1 (biofilm, AMP induction), *pelB::lux* (non-biofilm, AMP repression) or PAZHI3 (hyperbiofilm, AMP induction) for 24 h. After 24 h, orally infected flies from each of the three groups above and uninfected flies were nicked with PAO1 (acute infection), LB (sterile nicking) or not treated. Oral infection with PAO1, *pelB::lux* or PAZHI3 had no significant effect on the rate of fly survival during subsequent acute infection (nicking) with PAO1 (Figure 9).

To determine if oral PAO1 or PAZH13 biofilm infections altered *Drosophila* survival following subsequent oral infection with *pelB::lux,* the following experiment was performed. *Drosophila* were allowed to feed on PAO1, PAZH13, *pelB::lux* or a sucrose control for 24 h (primary infection), which is sufficient for biofilm formation to occur in the crop (Figure 1). After 24 h, all flies were transferred to new vials containing *pelB::lux* as the food source (secondary infection). Survival was monitored up to 14 days after the primary infection. Primary infection with PAO1 or PAZH13, followed by secondary infection with *pelB::lux* significantly increased fly survival compared to flies who were infected with *pelB::lux* for both the primary and secondary infection. Increased *Drosophila* survival following primary infection with PAO1 or PAZH13 was not due to failure of the secondary infecting *pelB::lux* strain to infect *Drosophila,* as *pelB::lux* tetracycline resistant colonies (the antibiotic marker of the *lux* transposon) were recovered (at ≥3.8×10^6^ CFU/fly or 76–99% of total bacterial load) from all secondary *pelB::lux* infected flies 5 days postinfection.

Primary oral infection with a biofilm-forming strain protected *Drosophila* from secondary oral infection with *pelB::lux*. Oral infection with a biofilm forming strain induced AMP gene expression, which may explain why increased fly survival was observed against secondary oral infection with *pelB::lux*. However the AMPs induced following oral infection may not be sufficient to alter *Drosophila* survival against subsequent acute infection. A possible reason for this is that AMP induction following biofilm infection is localized to the gut and does not protect *Drosophila* from death as a result of pricking and acute systemic infection. It is also possible that the pathology resulting from tissue damage following oral infection (Figure 4) may prevent *Drosophila* from responding to and coping with subsequent acute infection.

### Conclusions

*P. aeruginosa* infections are associated with the highest case fatality rate of all Gram-negative infections [67]. This is partly due to the ability of *P. aeruginosa* to resist antimicrobial therapy. One of the main evasion strategies used by *P. aeruginosa*, and other microbes, is the formation of multicellular, dense aggregates called biofilms. We have shown that specific antibiotic resistance mechanisms are induced in *P. aeruginosa* biofilms [4]. Biofilm infections are estimated to account for 65% of all bacterial infections [68]. While some studies have investigated the host response to *P. aeruginosa* infection [20], [69], [70], little is known regarding the bacterial and/or host factors involved in the pathogenesis of biofilm infections. The aim of this research was to develop a *Drosophila* infection model that enables biofilms to be intricately studied *in vivo*.

In this work we present evidence that oral infection of *Drosophila* by *P. aeruginosa* PAO1 resulted in biofilm formation in the *Drosophila* crop (Figure 1). We demonstrated that biofilms formed *in vivo* retain the typical characteristics of *in vitro* grown biofilms, including DNA and EPS staining (Figure 2) and increased resistance to antibiotics (Figure 6C). We also showed that biofilm infections resulted in significantly decreased numbers of bacteria disseminating to the hemolymph 2 days postinfection, and contributed to increased AMP gene expression in the fly (Figures 6, 8). Non-biofilm forming *pelB::lux* infections, on the other hand, resulted in decreased AMP gene expression in the fly, significantly increased numbers of bacteria disseminating to the hemolymph 2 days postinfection, as well as early and increased fly mortality (Figures 6–8). The increased virulence of the *pelB::lux* mutant was attenuated by co-infection of *Drosophila* with biofilm-forming and AMP-inducing strains PAO1 or PAZH13 (Figure 8D). Furthermore, primary infection with either of these AMP-inducing strains altered the survival kinetics of *Drosophila* from secondary oral infection with the more virulent *pelB::lux* but not from subsequent acute infection (Figure 9). In summary, we have developed a novel *P. aeruginosa* biofilm model of infection that can be used for studying both the bacterial and host response during infection. This model has the potential to significantly increase our understanding of the relationship between biofilms and the host during infection and also to tease out fundamental differences between the host response to biofilm and non-biofilm *P. aeruginosa* infections.

## Materials and Methods

### Bacterial strains and plasmids

*Pseudomonas aeruginosa* PAO1 and PAO1::p16Slux [63] were used as wildtype *P. aeruginosa* strains. *The pelB::lux* mutant is from a mini-Tn5-*lux* transposon mutant library that was previously constructed and mapped [48]. PAZHI3 is an *rsmA* mutant in the PAO1 background [61]. The plasmid pCHAP6656 encodes mCherry fluorescent outer membrane-anchored lipoproteins [42].

### *P. aeruginosa* oral infection of *Drosophila*

*Drosophila* were maintained routinely on medium containing corn meal, agar, sucrose, glucose, brewers' yeast, propionic acid, and phosphoric acid [71]. Infections were performed as previously described [25]. Mid-log phase LB cultures of *P. aeruginosa* were spun down and resuspended in 5% sucrose. Cultures were adjusted to an OD600 = 25 (2.5×10^10^ CFU per ml) in sucrose. The resuspended cells (0.12 mls) were spotted onto a sterile filter (Whatman) that was placed on the surface of 5 ml of solidified 5% sucrose agar in a plastic vial (VWR). The vials were allowed to dry at room temperature for approximately 30 minutes prior to addition of *Drosophila*. Because of the high concentration of bacteria on the feeding discs and the possibility of bacteria forming aggregates on the feeding discs over time, male Canton S flies (1–3 days old) were starved for 3 hours prior to being added to vials (10–14 flies per vial). This ensured that *Drosophila* fed heavily on *P. aeruginosa* within the first couple of hours. It is therefore unlikely that the *P. aeruginosa* strains on the filters had sufficient time to form biofilms prior to being eaten by *Drosophila* and causing an infection. Male flies were used as the infection lasts up to 14 days. During this time period females would have laid eggs, which if hatched, would interfere with the experimental results. Flies were anaesthetized by placing them on an ice-cold tile throughout the sorting and transferring process. Infection vials were stored at 26°C in a humidity controlled environment. The number of live flies to start the experiment was documented and live flies were counted at 24 hour intervals.

### Acute *P. aeruginosa* infection of *Drosophila*

Healthy 3 day-old male flies were used in the fly nicking assays according to a modified method of [20]. Flies were sorted following anesthesis on a cold tile. The male flies were nicked in the dorsal thorax with a 27.5-gauge needle (BD Biosciences), which was dipped in bacterial culture normalized to an optical density at 600 nm of 1.0 in LB broth. After nicking, 10–14 flies were placed into a vial of 5% sucrose agar and maintained at room temperature. Fly survival was monitored and recorded from 12 to 36 h postinoculation.

### Excision of gastrointestinal tract, crop and live cell imaging

Flies were sacrificed after which the inferior region of the abdomen was dissected under a dissecting microscope and the entire gastrointestinal (GI) system gently pulled through the resulting opening. The crop was separated from the rest of the GI system. For visualization of whole crop morphology, an Olympus OV100 intravital observation system was used and image analysis performed using Adobe Photoshop. For staining of bacteria and matrix components, crops were placed in phosphate buffered saline (PBS) and permeabilized with 0.1% Triton X100 for 15 mins. Following a wash step in PBS, crops were stained with fluorescent dyes of interest: 10 µg/ml FITC labelled HAA lectin (EY Laboratories, Inc) for exopolysaccharide, 80 µg/µl DAPI (Sigma) for *Drosophila* nuclei and DNA in the biofilm, and 1/40 (75 units) phallodin 488 (molecular probes) for F-actin. Crops were placed on a drop of PBS on a microscope slide, sealed with a coverslip and clear nail varnish and allowed to dry prior to viewing on a Leica DMIREB2 inverted, epifluorescence microscope. Crops were visualized using the 10, 40, 63 or 100x objective. Red, green and blue fluorescent images were merged using Adobe Photoshop.

### Quantification of biofilm formation in the crop

Image analysis using ImageJ was performed to identify and count the frequency of each individual cell, as well as small and large aggregates or biofilms present in the *Drosophila* crop. Using the ‘analyse particle’ function, the integrated density (sum of the grey values of the pixels in the object) of each event was measured. Data was organized into bins, depending on the integrated density of the cell/aggregate, counted and the frequency of each bin was calculated from 12 fields of view taken from at least 3 crops infected with either the wild-type PAO1 or the *pelB::lux* and *rsmA* mutants (Figure 4D). Bins were separated into three groups, individual cells, small microcolonies or large microcolonies, which had integrated density values >100,000, between 100,000–500,000 and >500,000, respectively. Images representing each of 3 strains are represented in Figure 5.

### Quantitative bacteriology from whole flies

Five infected live flies for each infection were crushed using a pellet pestle (Krackeler Scientific Inc.) in 300 µl PBS, serially diluted and plated onto *Pseudomonas* isolation agar (PIA; Difco) for PAO1 enumeration. To enumerate the CFUs in different regions of the fly, the GI systems of 3 flies was excised as previously described, crushed and plated. Flies with their GI tract removed were also pooled and plated. PIA plates were incubated at 37°C for 24 hours. Colonies were counted following incubation and CFU/fly was calculated.

### Hemolymph isolation

Hemolymph was isolated from 20 infected or uninfected flies in triplicate according to the method of Frydman, 2006 [72], yielding approximately 2 µl of hemolymph per replicate experiment. Hemolymph was serially diluted in PBS and plated on PIA agar to determine CFU per fly.

### Antimicrobial disc diffusion assay

*Drosophila* 5 days postinfection with PAO1 were crushed (5 flies per treatment) to give a predicted innoculum of 1×10^7^ CFU/ml. This was later verified by plate counts. Mock-infected flies consisted of planktonically grown PAO1 that was added to crushed uninfected flies prior to inoculation on PIA. This control was included to ensure any *Drosophila* product present in crushed flies did not alter the antibiotic resistance phenotype of PAO1. Plates were inoculated using a sterile swab. One µl of the following antibiotics were dispensed onto the agar plate: gentamicin (Gm) 30 µg/ml, polymyxin B (PxnB) 20 µg/ml, colistin (Coln) 20 µg/ml, ceftazadine (Ceft) 5.12 µg/ml and ciprofloxacin (CI) 10 µg/ml. Plates were incubated for 24 h at 37°C, after which zone sizes (mm) were determined. Zones of inhibition were measured for overnight cultures of planktonically grown PAO1, *Drosophila* 5 days postinfection with PAO1 and mock-infected PAO1.

### RNA isolation, reverse transcription and qPCR

Total RNA was extracted from five flies from each infection 24 hours postinfection using TRIzol (Invitrogen), as previously described [73] RNA was DNAsed using DNAfree (Ambion) and cDNA synthesized with a High Capacity cDNA synthesis kit (ABI Biosystems). 100 ng of cDNA was used as template in the Real-time PCR reactions. Custom TaqMan probes and for *diptericin* (Dm01841768_s1), *cecropin A1* (Dm02609400_s1) and *drosomycin* (Dm01822006_s1) and TaqMan Gene Expression Mastermix were used as recommended by the manufacturer (ABI Biosystems). RpL32 (Dm02151827_g1) was used as the constitutive control. Prokaryotic gene expression was measured using the iQ SYBR green supermix (Biorad) and bacterial specific primers to *pel*, *psl* and the 16S housekeeping gene (pelrtF 5′atcaagccctatccgttcct 3′, pelrtR 5′ aacggatggctgaaggtatg 3′, pslrtF 5′ agcagcaagctggtgatctt 3′, pslrtR 5′ggttgcgtaccaggtattcg 3′, 16SrtF 5′ gaaatccccgggctcaacctg 3′, 16SrtR 5′ccccacgctttcgcacctca3′). For quantitative RT-PCR (qRT-PCR), quantification and melting curve analyses were performed with an iQ5 (Bio-Rad) according to manufacturer's instructions. Each reaction is done in triplicate and standard deviations used to calculate a range of fold activation using the 2^ΔΔCt^ method [74].

### Statistical analysis

Survival curves were plotted and statistical analysis was performed using GraphPad Prism 5 software. 2-way ANOVA was used to calculate significant differences between PAO1 and mutant strains.

### *Drosophila* gene identification

The FlyBase gene identification numbers for *Drosophila* genes are as follows: Drosomycin FBgn0010381; Diptericin FBgn0034407; Cecropin A1 FBgn0000276.

## Supporting Information

Figure S1 Crops were harvested from twenty PAO1-infected flies 24 and 96 hours postinfection. The entire gut was removed and separated into crop alone, the gut (foregut, midgut and hindgut) and remaining fly body. Gene expression from the *lasI* promoter (chromosomally integrated at the mini-CTX neutral integration site [25]) was determined and used as a sensitive measure of localized bacterial load. Data are expressed as % of luminescence in each part of the fly body expressed as a percentage of the total luminescence measured.
(TIF)

Figure S2 EPS expression in different strains. (A) qRT-PCR analysis of *pel* and *psl* expression in PAZH13. Values are mean +/− SEM from triplicate qPCR experiments on two independently isolated RNA samples. (B) Congo red staining (Corrected A490) as a measure of EPS production in PAO1, *pelB::lux* and PAZH13. Values are mean +/− standard deviation of eight replicate cultures. * p<0.05.
(TIF)

Figure S3 *In vitro* biofilm formation and *in vivo* virulence of PAO1 *psl* mutant. (A) Biofilm formation as measured by crystal violet staining of total biomass adhered to pegs. (B) Kaplan-Meier survival curves during oral infection with PAO1, *psl*, or 5% sucrose control. The *psl* mutant was constructed by allelic exchange using the plasmid pMA8 [75] resulting in a 213 bp deletion in the *pslA* promoter region. Experiments were performed at least twice, each with a minimum of 50 flies and representative curves (mean +/− standard deviated) are shown.
(TIF)

Figure S4 Percentage colonization of wildtype and mutant strains for AMP gene expression studies. Percentage colonization at (A) 0 h, (B) 24 h and (C) 120 h postinfection. At relevant time points *Drosophila* (n = 6, from two independent experiments) were sacrificed, crushed and plated on PIA agar for enumeration of CFU. Data represented is mean +/− SEM.
(TIF)

Video S1 *P. aeruginosa* biofilms in the *Drosophila* organised in honeycomb-like structures.
(MOV)
