# Supplementary figures and images for: Drosophila melanogaster as an Animal Model for the Study of Pseudomonas aeruginosa Biofilm Infections In Vivo
Source: PLoS Pathog. 2011 Oct 6;7(10):e1002299. doi: 10.1371/journal.ppat.1002299 (PMC3188550; doi:10.1371/journal.ppat.1002299)

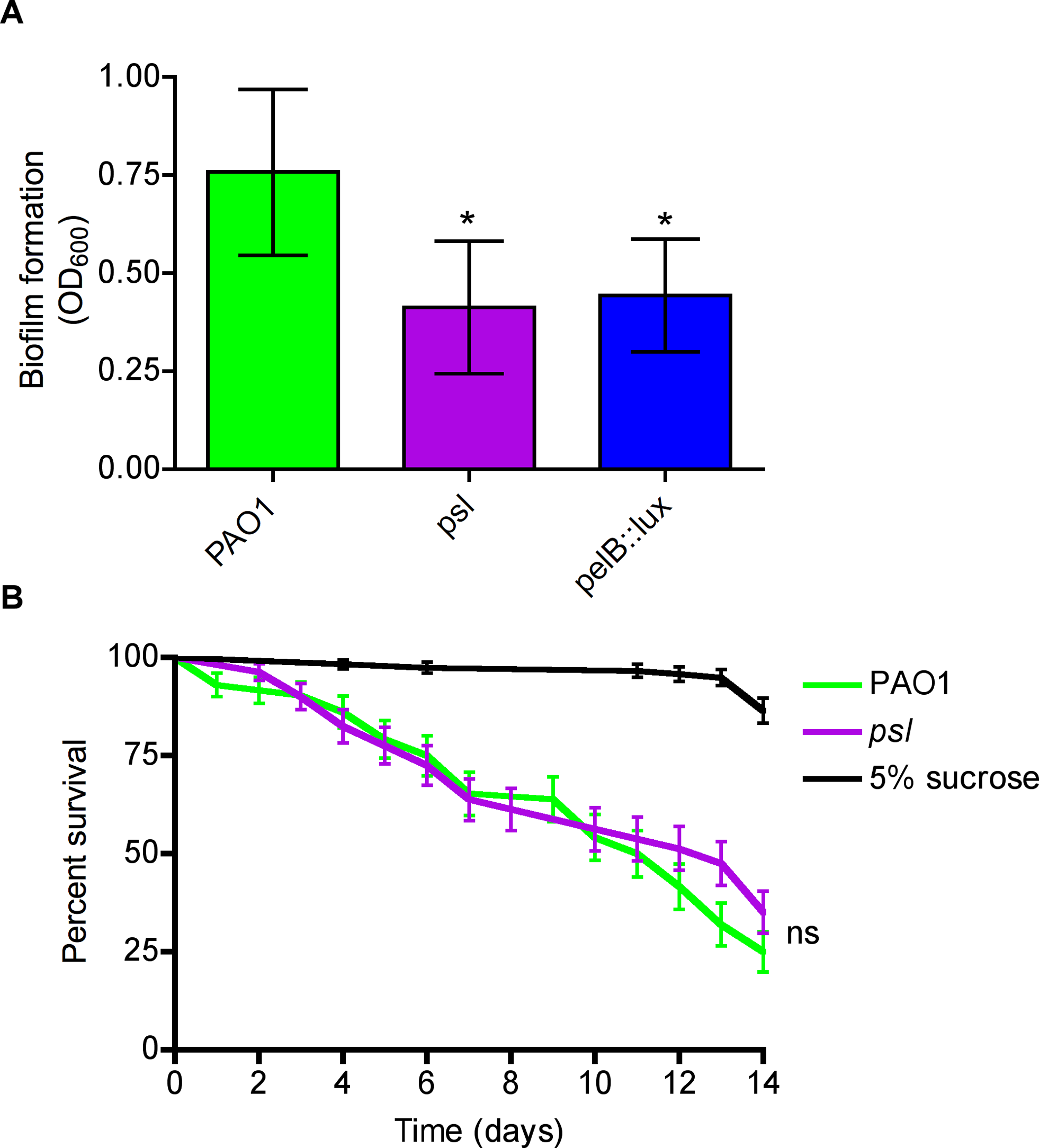

Supplement: Figure S3 — In vitro biofilm formation and in vivo virulence of PAO1 psl mutant. (A) Biofilm formation as measured by crystal violet staining of total biomass adhered to pegs. (B) Kaplan-Meier survival curves during oral infection with PAO1, psl, or 5% sucrose control. The psl mutant was constructed by allelic exchange using the plasmid pMA8 [75] resulting in a 213 bp deletion in the pslA promoter region. Experiments were performed at least twice, each with a minimum of 50 flies and representative curves (mean +/− standard deviated) are shown. (TIF) [file ppat.1002299.s003.tif]

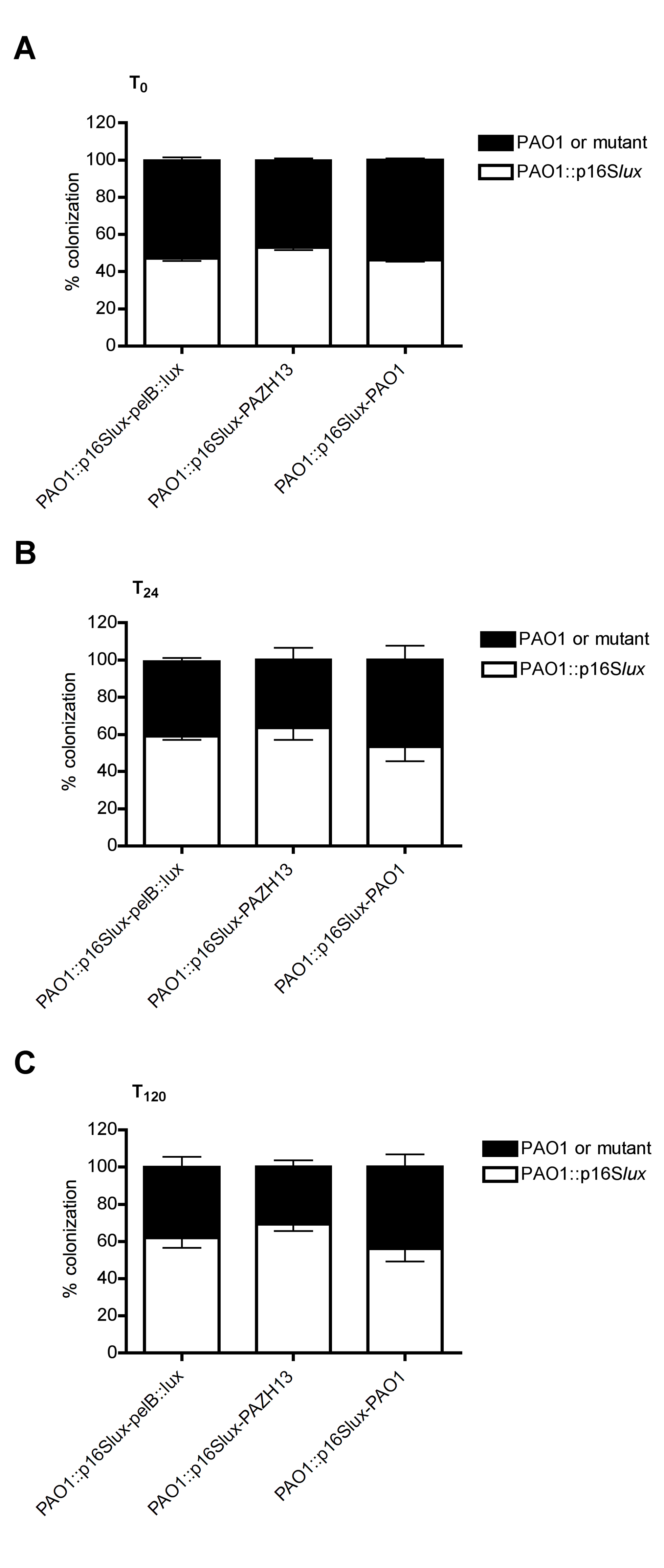

Supplement: Figure S4 — Percentage colonization of wildtype and mutant strains for AMP gene expression studies. Percentage colonization at (A) 0 h, (B) 24 h and (C) 120 h postinfection. At relevant time points Drosophila (n = 6, from two independent experiments) were sacrificed, crushed and plated on PIA agar for enumeration of CFU. Data represented is mean +/− SEM. (TIF) [file ppat.1002299.s004.tif]
